# ﻿The immatures of the New World treehopper tribes Acutalini Fowler and Micrutalini Haupt (Hemiptera, Membracidae, Smiliinae)

**DOI:** 10.3897/zookeys.1136.90525

**Published:** 2022-12-19

**Authors:** Stuart H. McKamey, Adam M. Wallner

**Affiliations:** 1 Systematic Entomology Laboratory, Agricultural Research Service, U.S. Department of Agriculture, c/o National Museum of Natural History, P.O. Box 37012, Washington DC 20013, USA c/o National Museum of Natural History Washington United States of America; 2 USDA-APHIS-PPQ Plant Inspection Station, 1500 Lower Road, Linden NJ 07036, USA USDA-APHIS-PPQ Plant Inspection Station Linden United States of America

**Keywords:** *Acutalis*, *Bordoniana*, immature stage, life history, *Micrutalis*, *Thrasymedes*

## Abstract

The nymphs of *Acutalis* Fairmaire, *Bordoniana* Sakakibara, *Thrasymedes* Kirkaldy, and *Micrutalis* Fowler are described and illustrated (*Bordoniana* and *Thrasymedes* for the first time). The nymphs of all four genera are exceedingly cryptic. The nymphs of some species lack scoli on the head and pronotum but all have paired scoli on the meso- and metathoracic nota and abdominal segments III–IX. Some species also have lateral rows of enlarged chalazae on the abdomen, and even large scoli ventrolaterally—the latter condition is unique within Smiliinae. The eggs are deposited in stems (not in exposed masses) and nymphs are solitary and not ant-attended. The fifth instar nymphs of Micrutalini range in length from 3.0–3.5 mm, much smaller than the fifth instars of most other treehoppers.

## ﻿Introduction

Adult treehoppers (Membracidae, Aetalionidae, and Melizoderidae) are well known for their expanded pronotum present in adults of more than 430 genera and 3,350 species (McKamey 1998 and recent additions). But in immatures, the pronotum is diminutive and accompanied by other structures, such as various arrangements of large spine-like structures (scoli) on the head and sometimes on the thoracic and abdominal segments, and enlarged setae with stalked or swollen bases (chalazae). We hypothesize that differences between adult and immature morphology may have evolved independently. Despite this wealth of potential diagnostic and systematically informative nymphal features, there have been few thorough descriptions of New World genera.

Besides the uniqueness of morphology of membracid nymphs, they also differ from nymphs of all other Auchenorrhyncha families in having the last visible abdominal segment (IX) fused ventrally, forming a tube containing the anal segments, which can be everted by the nymphs at will (McKamey and Brodbeck 2013). Behaviorally, membracid nymphs differ from those of most Auchenorrhyncha in being unable to jump (SHM pers. obs.).

The present paper is part of the larger effort to describe the immature stages of New World treehopper genera, which has so far covered the Caribbean genera *Antillotolania* Ramos, *Deiroderes* Ramos (McKamey and Brodbeck 2013), and tribe Quadrinareini (McKamey and Wallner 2022). The nymphs of the continental tribes Amastrini (McKamey et al. 2015) and Thuridini (McKamey and Porter 2016), and the membracine genus *Eunusa* Fonseca (McKamey 1992) have also been described. This work treats two more tribes of the New World subfamily Smiliinae: Acutalini and Micrutalini. The nymphs of all four genera described here are exceedingly cryptic (Figs 4–7, 42, 43). Both tribes occur throughout much of North and South America and the West Indies (McKamey 1998). The two tribes are grouped in this paper because of their small size and similar features in their nymphs and their adults. Adults of both tribes are commonly collected because they are often conspicuous with their black markings on the pronotum or wing veins (e.g., Figs 41, 43). In contrast, the nymphs of these tribes are rarely observed or collected.

Despite the scarcity of Acutalini and Micrutalini nymphs in collections, and their solitary nature and cryptic coloration and morphology, there has been some progress in nymphal descriptions. Quisenberry et al. (1978) illustrated and described, through a key, fifth instars of most genera of Membracidae present in Missouri, USA, including *Acutalis* and *Micrutalis*. They separated *Micrutalis* and *Acutalis* from other membracid genera based on the following features shared by both: paired median dorsal spines present on the abdomen but not on the head or pronotum, prothoracic tibia not dilated, anterior horn absent, and the outline of pronotum more or less rounded anteriorly. *Acutalis* was distinguished from *Micrutalis* based on a serrated dorsomedial line of the pronotum in *Acutalis* versus a smoothly rounded dorsomedial line of the pronotum and acute spines in *Micrutalis*. Tsai and Kopp (1981) described the life history, morphology, and phenology of *Acutalistartarea* (Say), illustrating the adults, eggs, and all instars. They reported that eggs were laid in clusters of 12–15 eggs each, inserted into the epidermal tissues of the host plant, with about 1/3 of the egg exposed, usually into the axis area of a leaf. They also reported that the nymphs were gregarious near the terminal portion of the plant of two Asteraceae: ragweed (*Ambrosiaartemisiifolia* L.) and China aster (*Callistephuschinensis* [L.] Nees, Asteraceae).

Deitz (1975) included three genera in Acutalini: *Acutalis* Fairmaire, *Euritea* Stål, and *Thrasymedes*. Generic additions since then include *Bordoniana* and *Cornutalis* (Sakakibara 1998, 1999a). Of these Acutalini genera, only nymphs of *Acutalis* have been described before now. The only host record for the genus *Cornutalis* is *Baccharis* sp., Asteraceae (Flórez-V. 2017).

Micrutalini only contains two genera: *Micrutalis* Fowler and *Trachytalis* Fowler. Several authors have contributed to our knowledge of micrutaline taxonomy and biology. For instance, Donald (1945) reported that *Micrutalis* sp. adults were “found in small numbers on *Cordiamacrostachya* (Jacq.) R. & S.” (Boraginaceae) in Trinidad. In the present study, nymphs and adults of two *Micrutalis* species were also collected on *Cordia*, one in Ecuador and another in Nicaragua (see Material examined under *Micrutalis*). Sakakibara (1976) described two new species, Deitz (1983) referred one species to *Rhexia* Stål, and Sakakibara (1999b) provided a synopsis of *Micrutalis*, including 42 species. Nixon and Thompson (1987) described and illustrated nymphs of *M.calva* (Say) and listed many hosts, listed below. Amaro (2009) reported *M.calva* on *Leucaenaleucocephala* (Lam.) de Wit (Fabaceae) in Cuba. Tsai and Brown (1991) provided a photograph of an *M.malleifera* Fowler nymph in a summary of pseudo-curly top virus in tomato (*Lycopersiconesculentum* Mill., Solanaceae). Flynn and Wheeler (2016) observed (but did not describe) nymphs of *Micrutalis*; they recorded adults of *M.pallens* Fowler on *Anisacanthusthurberi* [Torr.] A. Gray (Acanthaceae) but could not identify the observed nymphs because they were not reared to adults and there appeared to be two species on the host. Recently, however, Wheeler and Flynn (2021) described the nymphs of *M.discalis* (Walker) on mistletoe (Viscaceae), in general accordance with characters used in the aforementioned nymphal descriptions in this series.

In the present study, additional natural history information in provided as well as the descriptions of four genera of Acutalini and Micrutalini, two tribes of the New World subfamily Smiliinae. Nymphs of *Bordonia* and *Thrasymedes* have never been illustrated or described until now.

## ﻿Materials and methods

Preserved specimens were either collected by the first author or found in the U.S. National Collection. Vouchers of all examined nymphs and their associated adults are deposited in the National Museum of Natural History, Smithsonian Institution, in Washington DC (**USNM**). They were collected in Ecuador, Mexico, Nicaragua, Peru, the United States, and Venezuela.

Photographs of dried specimens were taken with a Canon 5Dsr camera with an adjustable 65mm lens. Photos were taken using Capture One Pro v. 10.1.2, 64 bit, build 10.1.2.23 imaging software, aided by CamLift v. 2.9.7.1. The specimen was illuminated using two adjustable Dynalite MH2050 RoadMax flash heads, each attached to a Manfrotto 244 arm. The light was diffused using a simple, lampshade-style cone of translucent paper between the specimen and light sources. After individual “slices” were photographed, they were compiled into a single, composite image using Zerene Stacker - USDA SI-SEL Lab Bk imaging system, v. 1.04, build T201706041920. Stacked images were enhanced and edited in Adobe Photoshop CSS Extended v. 12.0.

## ﻿Results

Key tzo 5^th^ instars of Acutalini genera (excluding *Euritea* and *Cornutalis*) and *Micrutalis*. Fifth instars differ from earlier instars in having a well-developed forewing pad that attains the posterior margin of the first visible abdominal segment (segment III) and usually overlaps part of the second visible segment (segment IV).

**Table:** 

1	Total length 3.5 mm or less	***Micrutalis***
–	Total length 4.3–8.5 mm	**2**
2	Abdomen laterally glabrous, not setose; dorsal scoli directed posteriorly (Figs 3–7)	***Acutalis***
–	Abdomen laterally densely setose (Figs 8, 12, 18); dorsal scoli directed dorsally or dorsoposteriorly	**2**
3	Abdominal tergum IX distinctly shorter than length of remaining abdominal segments combined (Figs 12, 13); abdominal terga IV–VIII with or without large scoli ventrolaterally (Fig. 14); head and prothorax with or without scoli	***Bordoniana***
–	Abdominal tergum IX as long as remaining abdominal segments combined (Fig. 18); abdominal terga IV–VIII without scoli ventrolaterally; head and prothorax with scoli	***Thrasymedes***

### ﻿Acutalini Fowler

Quisenberry et al. (1978) found features for distinguishing *Acutalis*, which is the only acutaline genus represented in Missouri, and those features were sufficient for that fauna. Considered within the larger context of Smiliinae, those features also apply to the Amastrini genera *Bajulata* Ball, *Erosne* Stål, *Harmonides* Kirkaldy, and some *Amastris* Stål (McKamey et al. 2015). But these features do not apply to the acutaline genus *Bordoniana*, which has abdominal scoli directed dorsally, nor the genus *Thrasymedes*, which has scoli on the head and pronotum, or even some other *Acutalis*, which have scoli on the head (1 pair) and pronotum (2 pairs) (Fig. 3). We also found that nymphs of Acutalini are solitary and not ant-attended, and we speculate that eggs are not laid in exposed masses but instead within host tissue. Tsai and Kopp’s (1981) report that the nymphs of *Acutalis* were gregarious near the terminal portion of ragweed host is interpreted here as a high population of solitary nymphs feeding at a preferred site with higher nitrogen. Nitrogen is recognized as an essential macronutrient for plant growth (Olas et al. 2019). Presumably, treehoppers feed on phloem, and plant nitrogen partitioning from source leaves to sinks occurs in the phloem (Tegeder and Masclaux-Daubresse 2017). Furthermore, Landrein et al. (2018) described a root-borne cytokinin signal that transduces nitrate availability to the shoot apical meristem within a matter of days and controls the stem cell population and, hence, meristem size and growth. In other words, meristems and young leaves have a higher concentration of nitrogen than other plant parts, and this may be why meristems are preferred feeding sites of many treehopper adults and nymphs.

Nymphs are unknown for the acutaline genera *Cornutalis* Sakakibara and *Euritea* Stål.

#### ﻿ Acutalis

Taxon classificationAnimaliaHemipteraMembracidae

Fairmaire

92BAA433-E869-5B11-835F-51768776BD53

Figs 1–7

##### Nymph diagnosis.

Body with full complement (9 pairs in total) of dorsal, short scoli from postmetopidium to last visible abdominal segment, and sometimes also 1 pair of scoli on head and 1 pair of scoli on premetopidium; metathoracic scoli directed forward, in opposite direction of abdominal scoli (backwards), low to tergal surface but not appressed; abdomen laterally with 3 rows of slightly enlarged chalazae but otherwise almost without setae.

##### Nymph description.

***Overall body*.** Fifth instar length 4.3 mm. Cross-section subtriangular; chalazae on thorax and abdomen, excluding those on scoli, sparse, almost absent; chalazal setae short; scoli parallel. ***Head*.** With simple conical scoli (except absent in *Acutalistartarea*), directed anterad, length relative size to basal width about subequal; chalazal bases long-stalked; compound eye surface with setae; frontoclypeus with dense setae; enlarged chalazae absent between eyes, but present in front of ventral margin of eye and also adjacent to central or dorsal margin of eye; frons extending over central margin of eye. ***Prothorax*.** Premetopidium scoli present (except absent in *Acutalistartarea*), directed anteriorly; postmetopidium scoli present, directed anteriorly; posterior extension of pronotum not surpassing anterior margin of metanotum; if present, premetopidial scoli length about 2–4× basal width; postmetopidial scoli length about 2–4× basal width. ***Mesothorax*.** Scoli bearing stalked chalazae; scoli directed dorsoanteriorly; forewing pad anterior costal margin straight; dorsal scoli length about 2–4× basal width; anterior basal side of scoli lacking cluster of enlarged chalazae; forewing pad surface chalazae absent; forewing pad costal chalazae present only only on base of costal margin; meso- and metathorax without lateral rows on enlarged chalazae. ***Metathorax*.** Scoli bearing stalked chalazae; scoli directed dorsoanteriorly; dorsal scoli length about 2–4× basal width. ***Legs*.** Chalazae of tibia on anterior and posterior lateral margins, absent or very few on dorsal surface; prothoracic tibia form subcylindrical. ***Abdomen*.** Terga III–VIII ventrolateral margins each with row of four or more enlarged chalazae; terga III–VIII dorsal scoli subequal in size to each other; terga III–VIII tallest dorsal scoli length 2–4× basal width; tergum IV dorsal scoli directed preapically dorsally, apically posteriorly but not appressed; terga III–VIII lateral rows bearing 3 rows slightly enlarged chalazae; lamellae absent; scoli bearing stalked chalazae. Segment IX: dorsal length subequal to combined length remaining visible abdominal terga; preapically with paired enlarged setae dorsally, with 1 pair dorsal scoli apically.

**Figures 1–7.**
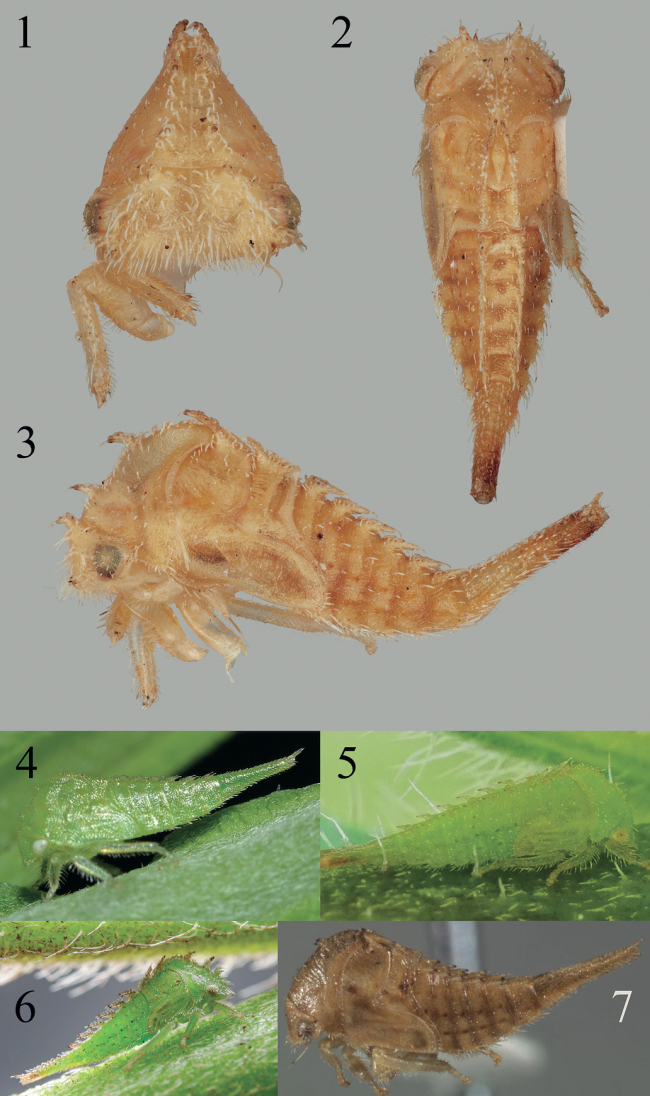
*Acutalis***1–3***Acutalisfusconervosa* Fairmaire from Chiapas, Mexico in anterior, dorsal, and lateral views, respectively **4***Acutalis* sp. from Durham, NC, courtesy of Margarita Lankford **5***Acutalis* sp. from Hoover, AL, courtesy of Vitaly Charny **6***Acutalis* sp. from Costa Rica, *ex*Asteraceae, courtesy of Kenji Nishida **7***Acutalistartarea*, courtesy of Mark Rothschild.

##### Material examined.

*Acutalisfusconervosa*, 1 adult, 2 nymphs, Mexico: Chiapas, 13 km S Pichucalco, 170 m alt., 17°26'38"N, 93°10'49"W, 2 November 2001, S.H. McKamey (USNM).

##### Note.

There is a difference between Quisenberry et al.’s (1978) illustration of *A.tartarea* (Say) and the specimen of *A.fusconervosa* Fairmaire figured here (Fig. 7); the latter has a small pair of scoli on the pronotum, lacking in Quisenberry et al.’s illustration. This difference cannot be attributed to developmental changes because both are fifth instars deduced from wing pad size. Quisenberry et al.’s (1978) illustration was redrawn from Matausch (1912b).

#### ﻿ Bordoniana

Taxon classificationAnimaliaHemipteraMembracidae

Sakakibara

ABDF0A5D-DC3C-5D82-B77D-5F9E977C714A

Figs 8–12 , 13–17

##### Nymph diagnosis.

Body densely setose; abdominal tergum IX distinctly shorter than length of remaining abdominal segments combined; abdominal terga IV–VIII sometimes with large scoli ventrolaterally; head and prothorax sometimes lacking scoli.

##### Nymph description.

***Overall body*.** Fifth instar length 5.1–6.5 mm. Cross-section subtriangular (except vertically depressed in *Bordoniana* sp. 2), chalazal dense on thorax and abdomen except scoli, obvious throughout body; chalazal setae long (expect short in *Bordoniana* sp. 1), scoli parallel (except splayed or divergent away from each other in *B.virescens*). ***Head*.** Scoli pair absent (except with simple conical scoli in *B.virescens*); scoli projection directed anterad in *B.virescens*; chalazal bases variable (see Remarks below); compound eye surface with setae; between eyes, enlarged chalazae variable (see Remarks below); scoli length about 2–4× basal width in *B.virescens*; enlarged chalazae present in front of ventral margin of eye and also adjacent to central or dorsal margin of eye (except enlarged chalazae absent in *Bordoniana* sp. 1.); enlarged chalazae adjacent to central or dorsal margin of eye present (except absent in *Bordoniana* sp. 1); frons extending over central margin of eye. ***Prothorax*.** Premetopidium scoli present (except absent in *Bordoniana* sp. 1); premetopidium scoli directed dorsoanteriorly; postmetopidium scoli absent; posterior extension of pronotum not surpassing anterior margin of metanotum but does not attain posterior margin (except surpasses posterior margin of metanotum in *Bordoniana* sp. 1); premetopidial scoli length relative to basal width variable (see Remarks below). ***Mesothorax*.** Scoli bearing tuberculate chalazae (except bearing stalked chalazae in *B.virescen*s); scolar direction variable (see Remarks below); forewing pad anterior costal margin sinuate (except straight in *B.virescens*); forewing pad chalazae short and dense, continuously covered (except densely covered in long setae in *Bordoniana* sp. 1); scoli length about 2–4× basal width (except scoli about as tall as basal width in *Bordoniana* sp. 1); anterior basal side of scoli lacking cluster of enlarged chalazae (except present in *B.virescens*); forewing pad costal chalazae present along entire costal margin (except present only at base of costal margin *B.virescens*); lateral rows, if present, with most medial row extending onto meso- and metathorax (except not extending onto thorax in *B.virescens*). ***Metathorax*.** Scoli bearing tuberculate chalazae (except bearing stalked chalazae in *B.virescens*); scoli directed dorsally or almost so (except directed posteriorly in Bordoniana sp. 1); scoli length about 2–4× basal width (except scoli about as tall as basal width in *Bordoniana* sp. 1). ***Legs*.** Tibia with chalazae present on both lateral margins and dorsal surface; prothoracic tibia form subcylindrical (except foliaceus in *Bordoniana* sp. 1). ***Abdomen*.** Terga III–VIII ventrolateral margins variable (see Remarks below); terga III–VIII dorsal scoli subequal in length relative sizes to each other subequal (except scoli size decreasing posteriorly in *B.virescens*); terga III–VIII tallest dorsal scoli length about 2–4× basal width; tergum IV dorsal scoli directed preapically variable (see Remarks below); tergum IV dorsal scoli directed apically dorsoposteriorly (except posteriorly in *Bordoniana* sp. 1); terga III–VIII lateral rows bearing 2 rows enlarged chalazae (except not manifested in *B.virescens*); lamellae absent (except present with lateral margins converging, apex pointed in *Bordoniana* sp. 2); lamellae (if lamella present) bearing chalazae marginally and dorsally; scoli bearing tuberculate chalazae (except bearing stalked chalazae in *B.virescens*). Segment IX: dorsal length subequal to combined length of segments V–VIII (except subequal to combined lengths of segments VI–VIII in *Bordoniana* sp. 1); preapically with dorsal surface irregularly covered in chalazae.

##### Material examined.

*Bordonianav*irescens Sakakibara, 1 adult, 1 nymph, Peru: Acobamba, July 1940, W.D. Funkhouser Collection (USNM); *Bordoniana* sp. 1, 1 adult, 1 nymph, Ecuador: Azuay, Baños, 2600 m alt., 23 May 1986, S.H. McKamey leg., lot# 05-23-23, 05-23-24 (USNM); *Bordoniana* sp. 2, 1 adult, 1 nymph, ECUADOR: Prov. Cañar. Ducur, via Cuenca-Guayaquil, 25-V-1986, ca 2520 m alt., S.H.McKamey leg., lot#86-0525-9, 86-0525-10 (USNM).

##### Remarks.

Substantial morphological variation was found among the nymphs of the *Bordoniana* species examined. Specifically, the head and pronotum may have or lack scoli on the head and pronotum, and one species of undescribed *Bordoniana* has large scoli venrolaterally on segments IV–VIII (Fig. 14); this is unique in Smiliinae and rare among other membracid nymphs (e.g., present in *Heteronotus* Laporte [figured in Deitz and Wallace 2010]).

We also discovered differences among the three species of *Bordoniana*. Head: chalazal bases tuberculate in *Bordoniana* sp. 1, short-stalked in *Bordoniana* sp. 2, and long-stalked in *B.virescens*; enlarged chalazae between eyes absent in *B.virescens*, present as a single pair in *Bordoniana* sp. 2, and present as pair of vertical rows in *Bordoniana* sp. 1. Prothorax: premetopidial scoli length relative to basal width about subequal to their basal widths in *Bordoniana* sp. 2, about 2–4× their basal widths in *B.virescens*, and scoli absent in *Bordoniana* sp. 1. Mesothorax: scoli directed dorsoanteriorly in *B.virescens*, dorsally or almost so in *Bordoniana* sp. 2, and directed posteriorly in *Bordoniana* sp. 1. Abdomen: terga III–VIII ventrolateral margins each with a single enlarged chalazae in *B.virescens*, with acuminate lateral extensions in *Bordoniana* sp. 2 (Fig. 14), and with a row of four or more enlarged chalazae in *Bordoniana* sp. 1; tergum IV scoli directed preapically dorsally or almost so in *B.virescens*, dorsoposteriorly in *Bordoniana* sp. 2, and posteriorly but not appressed in *Bordoniana* sp. 1. The most striking difference, described above, is that Bordoniana sp. 2 bears ventrolateral scoli, identical in placement but different in form to certain Centrotinae, Heteronotinae, Stegaspidinae, and *Procyrta* Stål (Darninae) (SHM pers. observ.). No other smiliine nymphs have ventrolateral scoli.

**Figures 8–12.**
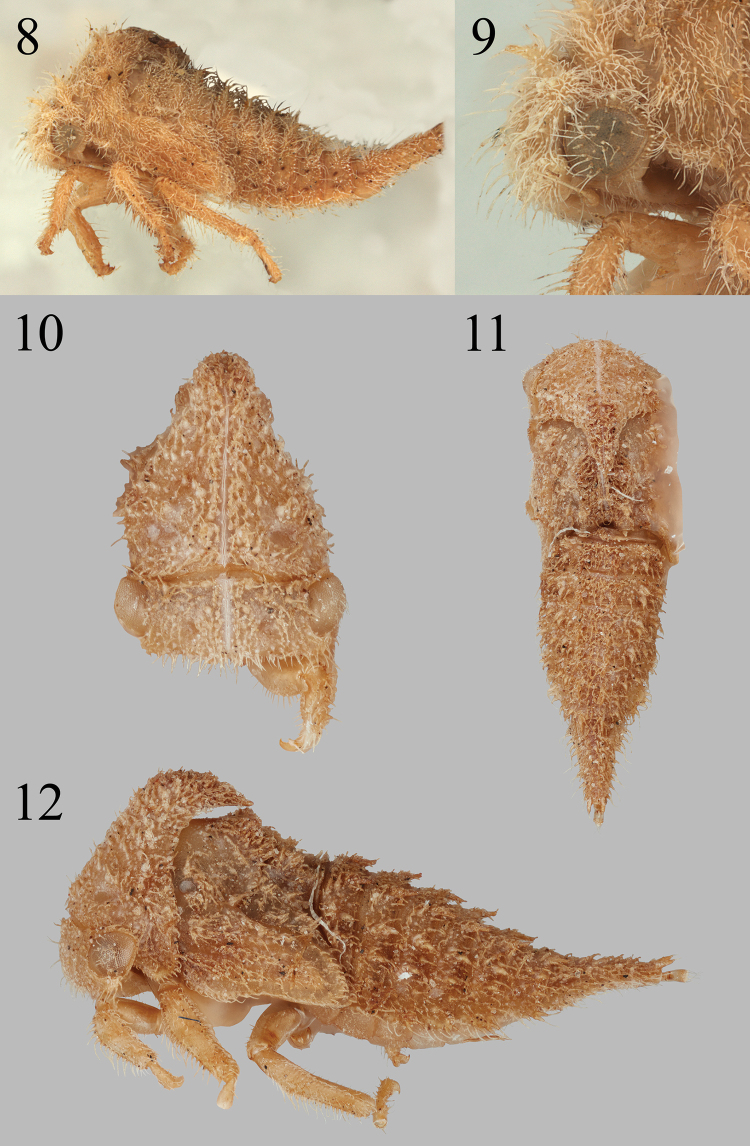
*Bordoniana***8, 9***B.virescens* Sakakibara in lateral view, habitus and detail of head and portion of thorax, respectively **10–12***Bordoniana* sp. 1 in anterior, dorsal, and lateral views, respectively.

**Figures 13–17.**
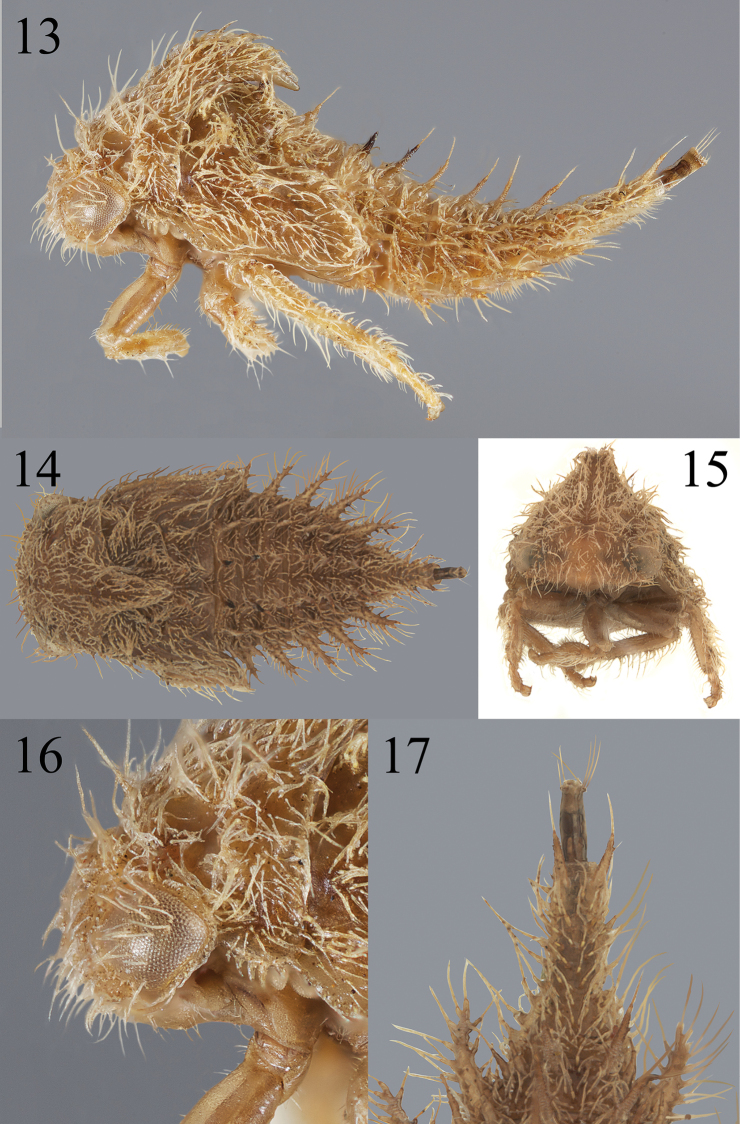
*Bordoniana* sp. 2 **13–17** nymph in lateral, dorsal, and anterior views, respectively **16** detail of head and portion of prothorax, lateral view **17** detail of abdominal segment IX (last visible segment), dorsal view.

#### ﻿ Thrasymedes

Taxon classificationAnimaliaHemipteraMembracidae

Kirkaldy

69836D02-47F8-5467-AD04-D349C860F7D5

Figs 18–22

##### Nymph diagnosis.

Body densely setose; with full complement of paired dorsal scoli from head to abdominal segment IX (12 pairs in total), though slender, not long and without stalked chalazae; abdominal segment IX as long as combined length of remaining abdominal terga; abdomen without scoli ventrolaterally.

##### Nymph description.

***Overall body*.** Fifth instar length 8.5 mm. Cross-section subtriangular; thorax and abdomen densely covered with chalazae, distinct throughout body; chalazal setae long; scoli parallel. ***Head*.** With simple conical scoli, directed anterad; chalazal bases long-stalked; compound eye surface with setae; enlarged chalazae between eyes present as pair of vertical rows; setae of frontoclypeus dense; scoli length about 5–7× basal width; enlarged chalazae in front of ventral margin of eye present; enlarged chalazae adjacent to central or dorsal margin of eye present; frons not extending over central margin of eye. ***Prothorax*.** Premetopidium scoli present, directed dorsoanteriorly; postmetopidium scoli present, directed anteriorly; posterior extension of pronotum surpasses anterior margin of metanotum, but does not attain1 its posterior margin; premetopidial scoli length about 5–7× basal width; postmetopidial scoli length about 5–7× basal width. ***Mesothorax*.** Dorsal scoli bearing stalked chalazae; scoli directed dorsoanteriorly and length about 5–7× basal width; anterior basal side of scoli lacking cluster of enlarged chalazae; forewing pad anterior costal margin straight; forewing pad surface densely covered by long chalazae; forewing pad costal chalazae along entire costal margin; lateral rows, if present, most medial row extending unto meso- and metathorax. ***Metathorax*.** Scoli bearing stalked chalazae; scoli directed dorsally or almost so; dorsal scoli length about 5–7× basal width. ***Legs*.** Chalazae of tibia present on both lateral margins and dorsal surface; prothoracic tibia form subcylindrical. ***Abdomen*.** Terga III–VIII ventrolateral margins each with 3 enlarged chalazae; terga III–VIII dorsal scoli length subequal to each other and bearing stalked chalazae; terga III–VIII tallest dorsal scoli length about 5–7× basal width; tergum IV dorsal scoli preapically directed dorsally or almost so, apically dorsoposteriorly; terga III–VIII bearing 1 lateral row of slightly enlarged chalazae; lamellae absent. Segment IX: longer than combined length of remaining abdominal terga, but shorter than length of rest of body; preapically with dorsal surface irregularly covered in chalazae.

##### Material examined.

*Thrasymedespallescens* (Stål): 39 adults, 1 nymph, 4 5^th^ instar exuviae, Mexico: Michoacán, Route 150, km 270, 40 km E Panindicuaro, 2150 m elev., 19°52'55"N, 101°24'45"W, 9 November 2001, S.H. McKamey leg. (USNM).

### ﻿Micrutalini Haupt

As for Acutalini, Quisenberry et al. (1978) gave distinguishing features for *Micrutalis* in Missouri, and those features were sufficient for that fauna. Their illustration, redrawn from Matausch (1912a), is consistent with a photograph of the live specimen (Fig. 23). The illustration by Nixon and Thompson (1987) shows the abdominal scoli more elevated than those in the specimen (Fig. 23) and also more than those in the illustration by Matausch (1912a).

**Figures 18–22.**
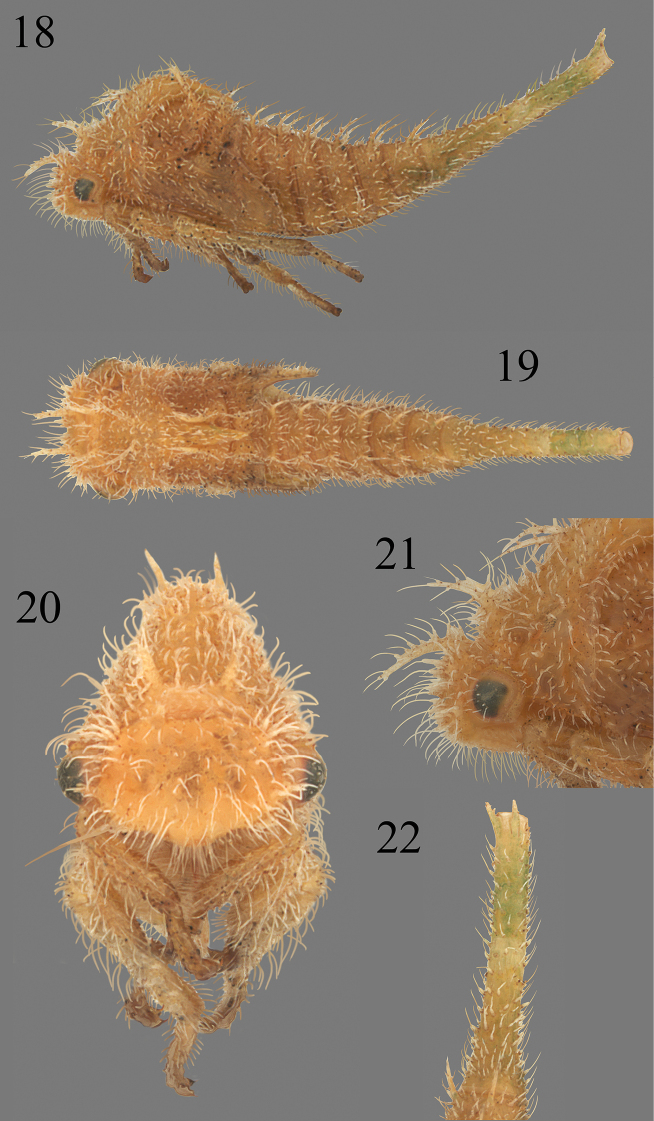
*Thrasymedespallescens* (Stål) **18–20** nymph in lateral, dorsal, and anterior views, respectively **21** detail of head and portion of prothorax, in lateral view **22** detail of abdominal segment IX (last visible segment), in dorsal view.

Considered within the larger context of Smiliinae, however, the features described by Quisenberry et al. (1978) and Nixon and Thompson (1987) are insufficient to distinguish them from the amastrine genera noted above (McKamey et al. 2015) and do not apply to all Neotropical *Micrutalis* species examined in our study. For example, Neotropical species of *Micrutalis* (except *Micrutalis* sp. 2) have scoli on all thoracic segments, not just the abdomen. Nymphs of *Micrutalis* are solitary and not ant-attended, and eggs are not laid in exposed masses.

In comparing morphology of adults and nymphs, we found more uniformity among *Micrutalis* adults than in their nymphs. Nymphs have yet to be discovered for *Trachytalis* Fowler, the only other micrutaline genus.

#### ﻿ Micrutalis

Taxon classificationAnimaliaHemipteraMembracidae

Fowler

93969165-9DF8-5A09-983D-6FCF638EDB2B

Figs 23–28 , 29, 30 , 31–34 , 35–37 , 38–43

##### Nymph diagnosis.

Fifth instar body length 3.0–3.5 mm; head and premetopidium lacking enlarged chalazae or scoli, postmetopidium with short scoli or enlarged chalazae; mesonotum to abdominal segment IX with small paired scoli; abdominal terga with 1 or 2 well-developed rows of enlarged chalazae or scoli; body densely setose, triangular in cross-section, not vertically compressed; abdomen lacking ventrolateral lamellae; wing pad costal margin linear or almost so; fused portion of abdominal segment IX directed posteriorly.

##### Nymph description.

***Overall body*.** Fifth instar length 3.0–3.5 mm. Cross-section subtriangular (except laterally compressed in *M.dubia* Fowler); chalazae on thorax and abdomen usually dense; chalazal setae long; no parts of body covered with wax-like substance; dorsal contour of abdomen in lateral view linear; scoli parallel; overall body in dorsal view elongate. ***Head*.** Lacking scoli; dorsal or anterior rounded protuberances absent; chalazal bases long-stalked (except tuberculate in *M.dubia*); chalazal setae simple, needlelike (except narrowly peltate in *M.dubia*) compound eye surface with setae; enlarged chalazae present or absent between eyes; setae of frontoclypeus scattered and sparse (except dense in *M.callangensis*); enlarged chalazae present in front of ventral margin of eye; enlarged chalazae present adjacent to central or dorsal margin of eye; frons extending over central margin of eye. ***Prothorax*.** Premetopidium lacking scoli; postmetopidium without dorsal paired structures or, if present (Fig. 33), with enlarged chalazae or small scoli directed dorsoposteriorly or dorsally then abruptly posteriorly; posterior extension of pronotum not surpassing anterior margin of metanotum, apex narrowly convex or acute; pronotal lateral margin rounded; postmetopidial scoli, if present, length about 2–4× basal width; metopidial sulcus not incised. ***Mesothorax*.** Dorsal structures consisting of paired scoli; scoli bearing stalked chalazae; scoli directed dorsoposteriorly or dorsally then abruptly posteriorly (except bluntly rounded in *M.callangensis*); forewing pad anterior costal margin form straight (except weakly sinuate in *M.dubia*); forewing pad surface chalazae sparse and with short setae (except densely covered in long setae in *M.callangensis*); scoli length about 2–4× basal width (except subequal to basal width in *M.callangensis*); anterior basal side of scoli lacking cluster of enlarged chalazae; forewing pad costal chalazae present only on base of costal margin (except along enture costal margin *M.callangensis*); lateral rows of abdomen with most medial row extending onto meso- and metathorax. ***Metathorax*.** Dorsal structures consisting of paired scoli; scoli bearing short-stalked chalazae; scoli directed dorsoposteriorly or dorsally then abruptly posteriorly; dorsal scoli length about 2–4× basal width (except subequal to basal width in *M.callangensis*). ***Legs*.** Tibia with chalazae present on both lateral margins and dorsal surface; prothoracic tibia form subcylindrical; metathoracic tarsal length subequal to pro- and mesothoracic tarsal length; all first tarsomeres distinctly shorter than second tarsomeres. ***Abdomen*.** Terga III–VIII ventrolateral margins lacking scoli but each with 2 enlarged chalazae (except with a single enlarged chalazae in *M.callangensis*); terga III–VIII with dorsal scoli present, subequal in size to each other (2–4× basal width). directed dorsoposteriorly or dorsally then abruptly posteriorly; terga III–VIII with lateral 1 or 2 rows of enlarged chalazae (Fig. 33) or manifested as scoli (in *M.callangensis*); abdominal scoli bearing stalked chalazae (except bearing tuberculate chalazae in *M.dubia*). Segment IX: distal half tubular in cross-section; dorsal length subequal to length of segment V–VIII (except subequal to combined length of remaining visible abdominal terga in *M.callangensis*); preapical dorsal surface irregularly covered with chalazae; dorsal structures at apex consisting of paired scoli; ventral extension subequal to dorsal extension; fused portion of segment IX directed posteriorly and distal to unfused portion; unfused portion distally not bifurcate.

**Figures 23–28.**
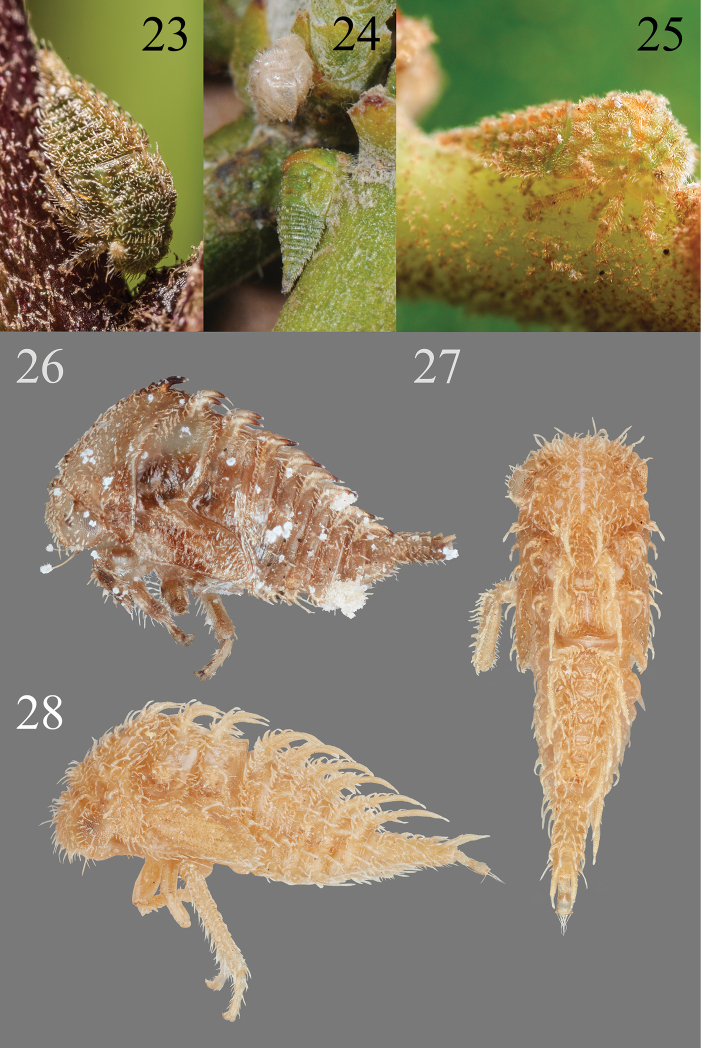
*Micrutalis* nymphs **23***M.calva* from Allison Park, Allegheny Co, PA, courtesy of John Rosenfeld **24***M.discalis* (Walker) on mistletoe from AZ, courtesy of Al Wheeler **25***Micrutalis* sp. from Costa Rica *ex Miconia calvescens* DC (Melastomataceae), courtesy of Kenji Nishida **26***Micrutalis* sp. from León, Nicaragua, lateral view **27, 28***M.dubia* Fowler, from Zona los Cinaros, Mérida State, Venezuela, in dorsal and lateral view, respectively.

**Figures 29, 30.**
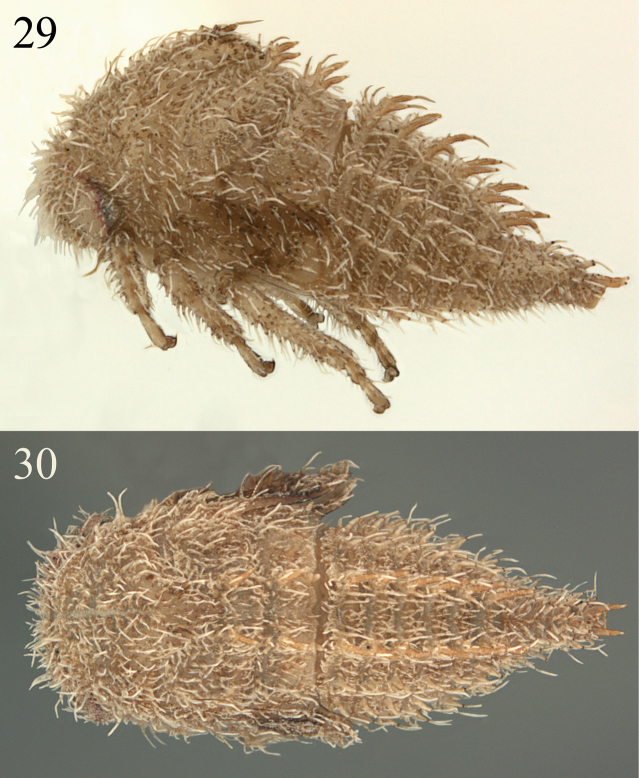
*Micrutalis* sp. 2 from Loja, Ecuador in lateral and dorsal view, respectively.

**Figures 31–34.**
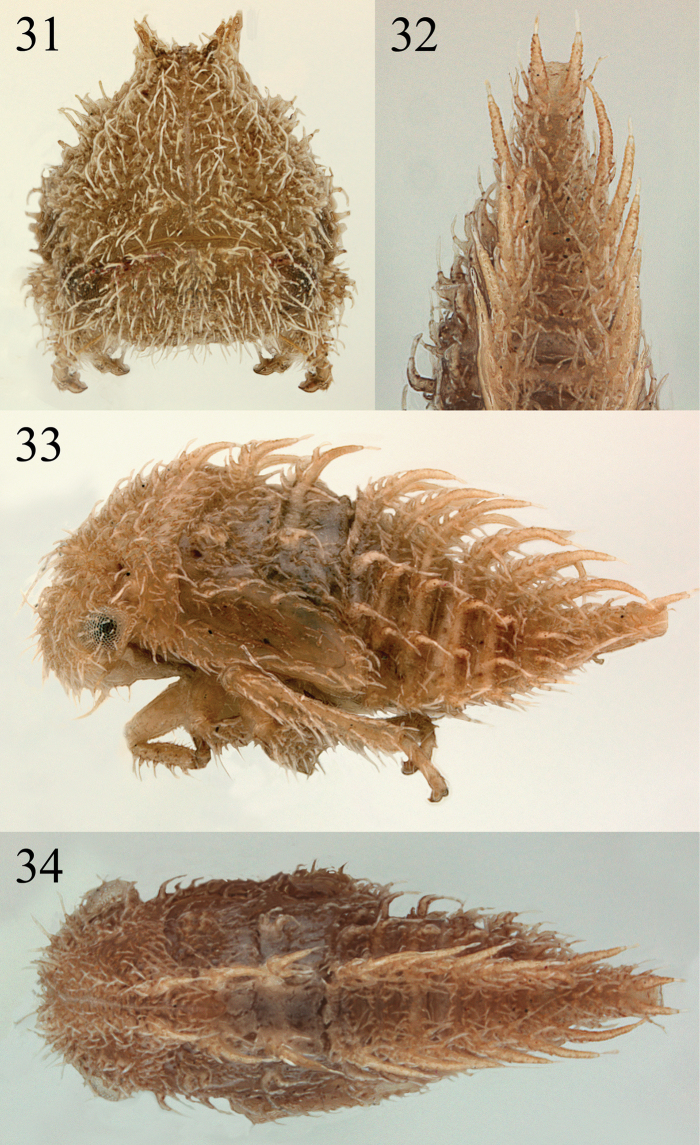
*Micrutalis* sp. **31** habitus anterior view **32** detail posterior abdomen in dorsal view **33, 34** habitus in lateral and dorsal views, respectively.

**Figures 35–37.**
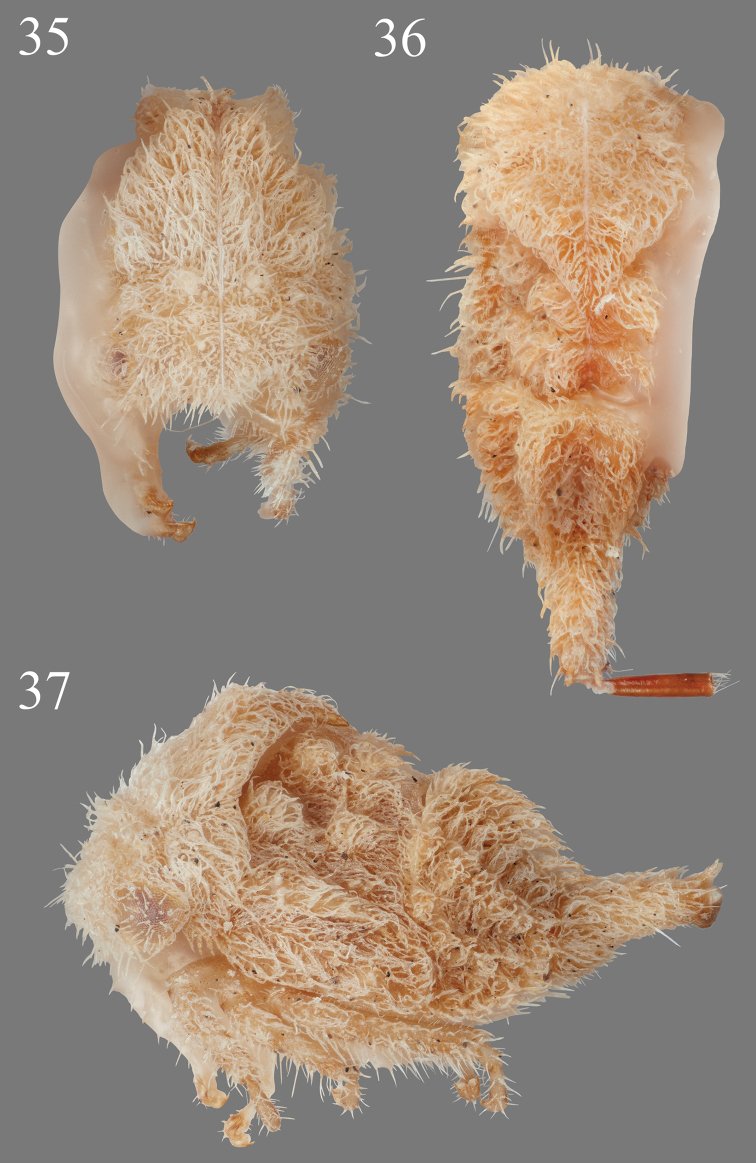
*Micrutaliscallangensis* Goding in anterior, dorsal, and lateral views, respectively.

**Figures 38–43.**
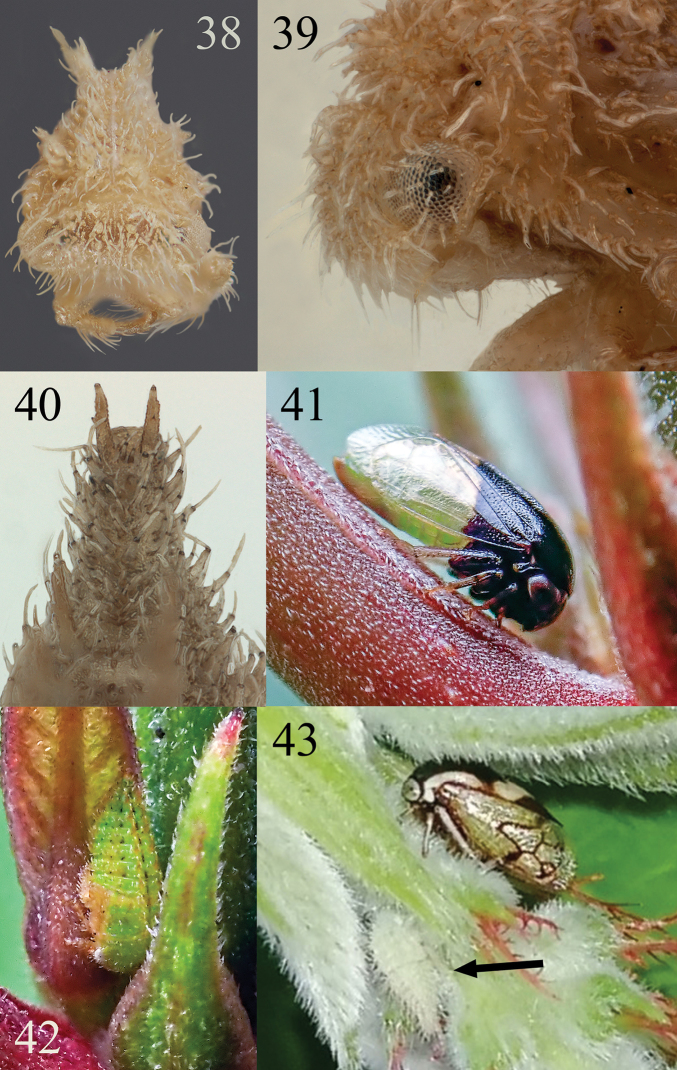
*Micrutalis***38–40***Micrutalis* sp. in anterior, detailed lateral head and portion of pronotum, and detailed abdomen, dorsal view, respectively **41, 42***Micrutalis* undescribed sp., adult and nymph from Costa Rica, ex *Hameliapatens* Jacq. (Rubiaceae), courtesy of Kenji Nishida **43***Micrutalis* undescribed sp., adult (upper right) and nymph (lower left, indicated by arrow) from San Juan, Bolivia by © Kozue Kawakami (CC BY).

##### Material examined.

*Micrutaliscallangensis*, 1 adult, 1 nymph, Ecuador: Cañar, Ducur, 25 May 1986, S.H. McKamey leg., lot # 86-0525-4, 86-0525-5 (USNM); *Micrutalis* undescribed species, 2 adults, 1 nymph, Nicaragua: Leon Finca N.I.L., 8 [October] 1989, J.M. Maes leg., ex *Cordia* sp. (USNM); *M.dubia*, 1 adult, 2 nymphs, Venezuela: Ed. Merida, Zona Los Cinaros, 58 km SW Merida, 24 July 1984, S.H. McKamey leg., lot #1008, 1009 (USNM). *Micrutalis* sp. 2, 2 adults, 2 nymphs, Ecuador: Loja, Loja, ca 2000 m alt., 30 May 1986, S.H. McKamey leg., lot #86-0530-7, 86-0530-8 (USNM); *Micrutalis* sp., 1 nymph (unassociated with adults), Mexico: Animal and Plant Health Inspection Service (APHIS) intercept APSCA191974874004 at San Ysidro, California, 15-VII-2019, ex *Dysphaniaambrosioides* (L.) Mosyakin & Clemants (Amaranthaceae; commonly known as espazote, Mexican tea, paico, and wormseed).

##### Hosts.

The great majority of *Micrutalis* species lack host information. Nevertheless, there are some host records in the literature and among specimens examined in this study. Nixon and Thompson (1987) reported that *M.calva* was polyphagous, with adults feeding on wormwood, soapwort, sycamore, redbud, ironweed, alfalfa, ragweed, sunflower, black locust, and honey locust. Nixon and Thompson (1987) also reported that nymphs have been collected on ironweed, ragweed, sunflower, and honey locust (*Gleditsiatriacanthos* L.); nymphs were collected on ironwood, ragweed, sunflower, and honey locust. The holotype of *M.henki*Sakakibara (1999b) was collected on *Luhea* [sic, for *Luehea*] *seemannii* Triana & Planch. Flynn and Wheeler (2016) recorded *M.pallens* on *Anisacanthusthurberi* [Torr.] A. Gray, Acanthaceae, but could not identify the *Micrutalis* species because the nymphs were not reared to adults. Wheeler and Flynn (2021) recorded *M.discalis* (Walker) from mistletoe (*Phoradendroncalifornicum* Nutt., Viscaceae). The *M.dubia* from Ecuador in our study was collected on *Cordia* sp., Boraginaceae. The APHIS intercepted nymph was *on Dysphania ambrosioide*s (L.) Mosyakin & Clemants (Amaranthaceae).

##### Remarks.

Although Micrutalini adults are distinguished by their wing venation and genitalia, the small size of the fifth instars of *Micrutalis* sets them apart from most treehoppers. The only New World treehoppers that rival their small size are some *Bolbonota* Amyot & Serville, *Eunusa* Fonseca, some Tragopini, Thuridini, Quadrinareini, some *Amastris* Stål, Centrodontini, Endoiastinae, *Deiroderes*, *Brachytalis* Metcalf & Bruner, *Brachybelus* Stål, and *Abelus* Stål. *Micrutalis* nymphs differ from the nymphs of all these small genera in one or more of the features listed above in the diagnosis of *Micrutalis*. In contrast to nymphs of *Micrutalis*, *Bolbonota* nymphs are covered with white wax-like exudate; *Eunusa* nymphs are covered with erect, stalked scoli and have the segment IX directed dorsally; nymphs of Tragopini, Thuridini, and Quadrinareini lack scoli entirely; Centrodontini and Endoiastinae lack setae, *Brachytalis* nymphs have the posterior margin of the metathorax mesally lengthened; and *Deiroderes* and *Brachybelus* nymphs have ventrolateally flattened abdominal lamellae. The only genus among these for which the nymphs are unknown is *Abelus*. We presume these resemble those of the closely related *Ischnocentrus* Stål, which have the costal margin of the wing pad notched. The most unusual *Micrutalis* species is *M.callangensis*, with its rounded meso- and metathoracic scoli, and its abdomen with lateral rows manifested as scoli rather than enlarged chalazae, and a proportionately longer segment IX.

## ﻿Conclusions

Considering the great variability that we have observed, morphological variation within Acutalini and within Micrutalini are underestimated. This situation is exacerbated by the absence of known nymphs for *Euritea* and *Cornutalis* (Acutalini) and *Trachytalis* (Micrutalini). This is especially the case for *Micrutalis*, for which only a few of the many species are known. For all genera in Acutalini and Micrutalini, we expect that more species will reveal more variability than accounted for here. In this respect it is like any taxonomic revision; it represents only the species studied and becomes outdated when more are available for examination.

## Supplementary Material

XML Treatment for Acutalis

XML Treatment for Bordoniana

XML Treatment for Thrasymedes

XML Treatment for Micrutalis
